# Nitrous Oxide, From the Operating Room to the Emergency Department

**DOI:** 10.1007/s40138-016-0092-3

**Published:** 2016-03-22

**Authors:** Christine Huang, Nathaniel Johnson

**Affiliations:** Department of Emergency Medicine, University of Arizona, 1609 N. Warren Ave., Tucson, AZ 85724-5057 USA

**Keywords:** Nitrous oxide, Pediatric emergency department, Procedural sedation, N_2_O

## Abstract

Nitrous oxide is a gas inhalation agent that has a long history of administration in procedures requiring analgesia and sedation. Although use may be limited by patient condition, patient comorbidities, and appropriate scavenging equipment, nitrous is a proven and safe tool for use in many health care settings—from the dental office to the operating room to the emergency department.

## Introduction: Procedural Sedation and Analgesia (PSA)

Physicians and medical care providers in general are charged with promoting health, as well as diagnosing and managing illness. The breadth of knowledge and skills required to manage multiple medical conditions, including emergency conditions, is vast and ever changing. In caring for our patients, knowledge and skill specifically in the management of pain and providing sedation are indispensable.

 Procedural sedation and analgesia (PSA) has become an integral tool for the physician in the diagnosis and management of disease processes and injuries. PSA is a technique utilized frequently by both anesthesiologist and non-anesthesiologists and involves the administration of a sedative or dissociative agent with or without an analgesic agent to induce a state that allows the patient to tolerate a painful and/or noxious procedure while maintaining spontaneous cardiovascular and respiratory functions. The levels of sedation for PSA can be divided into five different sub-groups [1] (Table 1).

**Table 1:** Levels of sedation

Sub-group	Description
Minimal sedation (anxiolysis)	*Patient response* the patient responds normally to verbal commands although cognitive functions and coordination may be impaired *Cardiovascular* functions remain unaffected *Respiratory* functions remain unaffected
Moderate sedation	*Patient response* the patient has a depressed state of consciousness but can respond purposefully to verbal commands with or without gentle tactile stimulation. This does not include reflexive withdrawal from a painful stimulus *Cardiovascular* functions remain unaffected *Respiratory* functions remain unaffected
Dissociative sedation	*Patient response* the patient is in a trance-like cataleptic state that is characterized by profound analgesia and amnesia *Cardiovascular* functions remain unaffected *Respiratory* functions remain unaffected
Deep sedation	*Patient response* the patient has a depressed state of consciousness and is not easily arousable but responds purposefully after repeated or painful stimulation *Cardiovascular* functions usually remain unaffected *Respiratory* functions may be impaired
General anesthesia	*Patient response* the patient has a loss of consciousness and is not arousable even with painful stimulation *Cardiovascular* functions may be impaired *Respiratory* functions are often impaired and the patient typically requires positive-pressure ventilation support

For example, PSA is frequently utilized in the treatment of the pediatric population to reduce the fear and anxiety as well as the pain experienced by children prior to and during procedures.

Characteristics of the ideal PSA agent would include

1. providing both sedation and analgesia,
2. having a quick onset of action and rapid recovery upon discontinuation,
3. preserving the cardiovascular and respiratory status of the patient,
4. imparting an amnestic quality.

Nitrous oxide possesses many of these properties, making it an ideal agent for PSA in the emergency department, as well as other medical arenas. In this article, we will review the history, mechanism of action, administration, applications, as well as the adverse effects of nitrous oxide.

## History

Nitrous oxide (chemical structure: N_2_O) was first isolated in 1772 by the English chemist Joseph Priestly and was originally given the name “dephlogisticated air” [2]. It was not until nearly two decades later that the Cornish chemist Humphry Davy would arrive at the Pneumatic Medical Institution in Bristol to conduct experiments on himself with N_2_O and discover its analgesic properties [3]. In 1800, he further went on to publish a book describing its potential role in medicine:

As nitrous oxide in its extensive operation appears capable of destroying physical pain, it may probably be used with advantage during surgical operations in which no great effusion of blood take place [4].

However the use of N_2_O for surgeries never took hold and it was instead relegated for recreational use as “laughing gas” into the early 19th century. It was briefly reintroduced into medical practice in 1844 when the American dental surgeon Horace Wells utilized the application of N_2_O to alleviate pain during tooth extraction. After a highly publicized failed experiment was conducted at the Massachusetts General Hospital, it then disappeared from the medical arena and was quickly overshadowed by the introduction of other notable inhaled anesthetics such as ether and chloroform [5].

Finally in 1863, almost a century after its initial discovery, Gardner Quincy Colton established the use of N_2_O in dentistry, solidifying it as a popular tool for painful dental procedures. Shortly thereafter, the use of N_2_O expanded into other medical fields, most notably obstetrics and surgery [6]. Since then, the administration of N_2_O has undergone numerous advances, increasing its safety and reliability. While the use of N_2_O as an adjunctive induction agent for anesthesia in dentistry and the operating room has been well established, it was only relatively recently that the use of N_2_O has emerged as a valuable tool for PSA in the emergency department/acute care setting.

## Mechanism of Action

Nitrous oxide is a colorless and mostly odorless gas that is stable at room temperature and ambient pressure. It is heavier than air. When inhaled, it has been demonstrated to have both anesthetic and analgesic properties. While the exact mechanism of action of N_2_O remains to be completely elucidated, there are several existing theories that describe how it exerts its effects. Unlike other well-known anesthetics, such as propofol and etomidate, which act directly on the inhibitory γ-aminobutyric acid (GABA) type A receptors, N_2_O has been shown to modulate a different target. The prevailing theory suggests N_2_O provides its anesthetic properties through the non-competitive inhibition of the excitatory NMDA subtype glutamate receptors [7].

In contrast, the mechanism by which N_2_O exerts its analgesic effects is much better understood. Research indicates that N_2_O induces endogenous opioid peptide release by binding to opioid receptors located in the periaqueductal gray matter and noradrenergic of the brainstem. This results in the release of opioid in the brainstem, which in turn inhibits the GABA neurons thereby removing the tonic inhibition on the descending noradrenergic inhibitory pathways. This dis-inhibition of the noradrenergic neurons in the brainstem modulates nociception by releasing norepinephrine into the spinal cord thereby inhibiting pain signaling [8, 9].

The potency of nitrous oxide is dependent on the minimum alveolar concentration (MAC). The minimum alveolar concentration of an inhaled anesthetic is defined as the expired concentration or dose of an inhalational agent required to suppress movement in 50 % of patients in response to a surgical stimulus. Thus the lower the MAC, the more potent the agent. There are several factors that can influence the MAC of a particular agent such as age, addition of adjunctive medications, and pre-existing patient conditions. The MAC_50_ is 104 % for N_2_O, thus making it a poor single agent for general anesthesia. However, when administrated at sub-MAC concentrations in combination with an analgesic, it acts as an effective anxiolytic ideal for PSA [10].

## Administration

Nitrous oxide is typically stored under 750 psi as a vapor overlying a liquid in a color-coded tank; in the United States, the standard tank color is blue. N_2_O is neither flammable nor explosive, but it is capable of supporting combustion and therefore open flames should not be allowed near the storage tanks.

N_2_O can be administered as a 30–70 % N_2_O–oxygen mixture by a demand-valve mask or mouthpiece held by the patient (self-administration). It is typically administered as a 50–50 % or 70–30 % N_2_O–oxygen mixture, with at least 30 % oxygen to avoid hypoxemia. A 30 % N_2_O concentration may be less than effective for PSA, especially in children. Administration of high concentrations of N_2_O (50–70 %) for PSA has been shown to be safe with no significant difference in the rate of adverse events between the two groups [11].

Although N_2_O is considered a mostly odorless gas, the tubing and mask that comprise the delivery system often times have an unpleasant odor that is disturbing to pediatric patients. Therefore, the common form of practice is to coat the inside of the mask with a liquid that produces a vaporized scent that is pleasing to the child, such as strawberry or bubblegum.

## Benefits of N_2_O for PSA

There are many attractive qualities of N_2_O that make it an appealing agent for PSA, particularly in the pediatric emergency department [12]:

1. N_2_O is known to be an excellent anxiolytic that can be administered without the need for intravenous access. Particularly in the pediatric population, sometimes the means to a cure (e.g. injection of an anxiolytic) can often times be viewed as worse than the actual cure itself (e.g. suturing a simple forehead laceration). Thus, in caring for a fearful toddler, N_2_O may be a far superior choice when compared to other anxiolytics at our disposal, particularly those that require intravenous access or irritating intranasal administration. N_2_O even has advantage over oral medications that typically have a delayed onset of action.
2. Due to its low solubility in the blood (blood–gas partition coefficient = 0.47), this allows for rapid onset of action in the brain (30–60 s) as well as rapid clearance through the lungs shortly after discontinuation [13]. In a busy emergency department, decreasing the length of stay for patients requiring procedural sedation for painful procedures is of benefit.
3. It provides a minor amnestic effect. Children who have been sedated with N_2_O often have little recollection of the painful elements of the procedure [14].
4. It has a long history of having an excellent safety profile with few reported cases of adverse events. The most frequently reported adverse reaction is nausea and vomiting. There are few reported cases of serious injury from appropriate N_2_O administration. There are no reported allergies to N_2_O and it has not been shown to be associated with malignant hyperthermia [15]. There have been several case reports of nitrous oxide-induced myelopathy [16], polyneuropathy [17, 18], and seizure activity [19] reported. Some of these cases are associated with higher exposures and/or abuse.
5. It is easily transportable. The units that are manufactured for supplying nitrous oxide in the emergency department are small and portable making it easy to store and transport between patient rooms.
6. The use of N_2_O at PSA concentrations has been shown to have minimal effects on the hemodynamic status, spontaneous respiration, cerebral blood flow, and protective airway reflexes of the patient [13]. This makes it a remarkably safe drug to use in emergency situations where patients may already present with underlying physiologic compromise.
  - *Cardiovascular* Although N_2_O has been reported to decrease myocardial contractility in vitro, it also simultaneously stimulates catecholamine release by the sympathetic nervous system thereby leaving cardiac output, blood pressure, and heart rate relatively unchanged in vivo.
    CO = SV × HR
    where CO is cardiac output, measured in L/min
    SV is stroke volume, measured in L/beat, and
    HR is heart rate, measured in beats/min.
  - *Respiratory* N_2_O has the effect of increasing the respiratory rate and decreasing the tidal volume thereby maintaining relatively unchanged minute ventilation.
    $$\dot{V} = V_{\text{T}} \times f,$$
    where$\dot{V}$ is minute ventilation, measured in L/min, *V*_T_ is tidal volume, measured in L, and *f* is respiratory rate, measured in breaths/min.
  - *Cerebral blood flow* N_2_O causes a small increase in cerebral blood flow and volume thereby producing a mild elevation in intracranial pressure. It may also slightly increase cerebral oxygen consumption.
    CBF = CPP/CVR and
    CPP = MAP − ICP
    where CBF is cerebral blood flow, CPP is cerebral perfusion pressure, CVR is cerebrovascular resistance, MAP is mean arterial pressure, and ICP is intracranial pressure.

## Applications of Nitrous Oxide

### Dentistry

As mentioned earlier, N_2_O was first popularized in the 1860s as an anxiolytic and analgesic tool for performing simple dental procedures such as tooth extraction. Remarkably, it has endured through time and is still used in modern dentistry as one of the most frequently employed inhalational sedatives for pediatric dental procedures. The benefits of N_2_O in dentistry are threefold: (1) anxiolysis, (2) depressed gag and swallow reflexes, and (3) analgesia [15]. Nitrous oxide is more commonly used today as an adjunctive anxiolytic, as there is a wide variety of oral sedative medications that can be taken prior to arrival for their dental procedure. In addition, pre-medication with oral sedatives such as midazolam has been shown to increase the depth sedation when combined with N_2_O during the procedure [20].

### Operating Room

Surgeons and anesthesiologists were also among the first to utilize inhalational gases for general anesthesia in patients undergoing surgical operations. Nitrous oxide was one of the first inhalational anesthetics observed to be beneficial in the operating room. However, in modern day use it has taken a lesser role as compared to other more potent inhalational anesthetics. However, it is still widely used in operating rooms today for several reasons: (1) it does not require IV access, (2) it has a rapid on/off effect, (3) it is relatively inexpensive as compared to other inhalational anesthetics, and (4) it does not have significant emergence issues [12].

### Emergency Department

Many procedures that are commonly performed, frequently in the emergency department, have a tendency to incite a high level of anxiety, particularly in the pediatric population. Addressing pain and anxiety is not only benevolent (for both the patient and parents) but also necessary to help facilitate the safe and efficient execution of various emergency department procedures.

Several studies have examined the efficacy and safety of N_2_O in PSA. These studies look at the use of N_2_O during PSA for a variety of procedures ranging from wound examination to minor surgery. In a review article published in 2013, seven out of eight articles that examined the use of N_2_O in PSA noted favorable outcomes [21]. The one article that did not show a favorable outcome had a significant percentage of patients (45 %) who reported pain scores greater than 50 (on a scale of 0–100). The same study, however, also reported a high percentage (93 %) of parental satisfaction with the procedure [22].

PSA with N_2_O has also been examined in the setting of a single specific procedure. N_2_O has been demonstrated to be an extremely effective anxiolytic; however, due to its low potency as an analgesic agent, it is rarely used as a single agent. Often times, it is paired with either a local nerve block or systemic analgesia to provide maximum pain relief before and/or during a procedure.

### Venipuncture

Several studies have examined the efficacy of N_2_O for anxiolysis and analgesia for venipuncture. These studies have shown that it is both effective as a single agent [23] and in combination with topical analgesic creams such as EMLA [24, 25].

However, in a cost-effectiveness study comparing several methods of analgesia for intravenous cannulation, needle-free jet injection of lidocaine had the best cost-effectiveness ratio, better than N_2_O [26]. Therefore, the widespread use of N_2_O solely for the purpose of venipuncture may not be as favorable in the emergency department.

### Laceration Repair

Laceration repair is a frequent reason for pediatric emergency department visits. Wound closure is not only cosmetically beneficial, but also helps accelerate the healing process and prevent further wound contamination. There are several studies that examine the efficacy of N_2_O for primary wound closure [27–30]. They conclude that N_2_O is not only effective at decreasing anxiety [30] and pain [28], but that N_2_O is actually preferable over other sedatives such as oral midazolam [29] or intravenous ketamine [27] given its fast induction time and quick recovery time. Furthermore, N_2_O was shown to have less adverse effects, such as nausea and vomiting, as compared to oral midazolam [29] or intravenous ketamine [27].

### Fracture Reduction

Fracture reduction is one of the most painful procedures commonly performed in the Pediatric Emergency Department. Even under the best PSA conditions, children often react to the aggressive reduction maneuvers required to realign fractured bones. Nitrous oxide has been demonstrated to be safe to administer, although less effective as a single agent for analgesia during fracture reduction [31]. However, in combination with a hematoma block, N_2_O has been shown to be effective in providing adequate PSA for fracture reductions [32, 33]. Furthermore, in one study comparing N_2_O + hematoma block versus ketamine + midazolam, N_2_O + hematoma block was shown to have fewer adverse effects and a significantly quicker recovery time [33].

### Other

There have been several additional studies that have examined the use of N_2_O in the setting of other painful and/or uncomfortable procedures. As may be reasonably inferred by its efficacy for intravenous cannulation, N_2_O has also been shown to provide adequate PSA for other emergency department procedures that involve needle punctures such as intra-articular injections [34] and lumbar puncture [35]. Nitrous oxide also has been shown to be effective in reducing pain and anxiety during limited burn debridement [36]. Other procedures that have been successfully performed with the aid of N_2_O include abscess drainage, foreign body removal, urinary catheterization, wound examination, and joint aspiration [21].

In addition to PSA, N_2_O may also be utilized to manage pre-existing pain. There have been only a few studies that have examined the use of N_2_O for pain management. One study demonstrated that N_2_O may have some benefit for the short-term management of migraines [37] as well as to enhance the analgesic effect of morphine in vaso-occlusive crisis in the sickle cell patient [38].

To our knowledge, there have not been any published papers examining the use of N_2_O for uncomfortable or unsettling examinations that may need to be urgently conducted in the emergency department such as child abuse exams, ophthalmological exams, and diagnostic imaging. However, given that these exams are mostly anxiety provoking rather than painful, administration of N_2_O may be beneficial in facilitating the prompt execution of these types of examinations while minimizing anxiety in the pediatric patient.

### Pre-hospital Setting

Many traumatic patients arrive to the emergency department via pre-hospital emergency medical services (EMS). In addition to stabilizing the patient, part of the EMS objective is to provide initial pain management. The use of N_2_O has been proposed and studied in the pre-hospital setting. In a double-blind multi-center study conducted in France, the investigators were able to show that pre-hospital administration of a fixed 50:50 ratio of N_2_O:O_2_ was effective in management of traumatic pain in the field [39]. However, further studies are needed to further assess the safety of its use in the pre-hospital setting.

## Side Effects

The administration of nitrous oxide is relatively safe, with few patients experiencing any adverse events. Some of the most commonly reported negative side effects of N_2_O include vomiting, nausea, dizziness, headache, tingling, and euphoria [14]. The risk of nausea and vomiting after N_2_O administration increases with the duration of sedation [40]. However, in cases where N_2_O was administered for <15 min, the rate of nausea and vomiting were 1 to 1.6 % [41]. Furthermore, the fasting status of the patient prior to the procedure does not appear to have a significant effect on the frequency of adverse events [42].

There are very few instances of major complications reported with the use of N_2_O, such as oxygen desaturation, aspiration, or bradycardia. The rate of major events was mainly affected by (1) age and (2) the use of adjunctive medications such as benzodiazepines [43]. However, despite its good track record, there was a case report of laryngospasm and with apparent aspiration with administration of N_2_O as a single agent published in 2015. The authors highlight the fact that although generally safe, N_2_O should still be administered in settings where airway emergencies can be quickly managed [44]. Diffusion hypoxia can occur with the use of nitrous oxide especially when self-administration of the nitrous oxide ceases or at the end of the procedure. Diffusion hypoxia refers to the decrease in alveolar oxygen tension when room air is inhaled at the conclusion of nitrous oxide administration due to nitrous oxide diffusing out of the blood and diluting the alveolar oxygen. This is why continuous pulse oximetry is recommended when using nitrous oxide. [24] (Table 2)

**Table 2:** Nitrous oxide: side effects and contraindications

More common side effects
Nausea/vomiting
Dizziness
Headache
Tingling
Euphoria
Diffusion hypoxia

Contraindications—any “trapped air” in the body
Examples of “trapped air” include
Pneumothorax
Chronic obstructive emphysema (COPD)
Pneumocephalus
Intraocular air bubbles
Middle ear effusions
Air embolism
Bowel obstruction
Decompression sickness
Bullous or emphysematous lung disease (such as advanced cystic fibrosis)

There has been a recent report of one case report of laryngospasm with aspiration with the administration of nitrous oxide as a sole agent

## Contraindications

As mentioned earlier, N_2_O has a low solubility in the blood and this characteristic accounts for its rapid onset of action in PSA. However, essential to the evaluation of its safety, it is important to note that N_2_O is 35 times more soluble than nitrogen (the principal component of air) in blood. Thus, when N_2_O is inhaled, it will diffuse into air-filled spaces faster than nitrogen can be reabsorbed into the bloodstream. In enclosed cavities of the body, this may result in the rapid expansion of gas resulting in injury from increasing pressure [13]. Examples of conditions where this may be of concern include chronic obstructive pulmonary disease or any other bullous or emphysematous lung disease including advanced cystic fibrosis, pneumothorax, pneumocephalus, intraocular air bubbles, middle ear effusions, air embolism, bowel obstruction, and decompression sickness. The magnitude of the pressure increase and volume expansion caused by N_2_O depends on several factors: (1) the *P*_a_ of nitrous oxide, (2) blood flow to the air-filled cavity, and (3) duration of exposure. Nitrous oxide has been demonstrated to increase the volume of a pneumothorax [45]. As demonstrated in an animal model, administration of 75 % N_2_O doubles the volume of a pneumothorax in 10 min [10]. However, another animal study from 1995, though showing an increase in pneumothorax size, showed essentially no clinical effect [45]. (Table 2).

## Toxicity

The most notable toxicity reported from the inhalation of nitrous oxide is the irreversible inactivation of cobalamin (vitamin B_12_). Vitamin B_12_ serves as an important co-enzyme (Co-B_12_) in two metabolic processes in the human body: (1) methylmalonyl-CoA-mutase and (2) methionine synthase. Inhibition of the methionine synthase enzyme results in decreasing levels of hematopoietic functions and increasing levels of homocysteine concentrations [46, 47]. Thus, chronic use of nitrous oxide can theoretically result in hematologic problems, bone marrow suppression, and CNS toxicity. However, despite its inhibitory effect on vitamin B_12_, with limited exposure (up to 8 h), there has been no clinical sign of megaloblastic anemia seen in pediatric patients undergoing lengthy surgical procedures [48].

Given the potential effects from long-term exposure to nitrous oxide, hospital personnel who work in areas where nitrous oxide is being utilized are in theoretic danger. Environmental exposure to nitrous oxide in the emergency department is one reason for scavenger devices and proper ventilation to be installed where N_2_O is being utilized. In areas where N_2_O is administered with proper a scavenger device, 0 ppm of N_2_O is detected in the surrounding environment [49].

## Abuse Potential

The term “laughing gas” is a common layman’s term for nitrous oxide, given its ability to create a sense of euphoria in the user. In addition, as noted earlier, it has an opioid-like effect, which accounts for its analgesic properties. Nitrous oxide can be found in a variety of readily available items such as whip-cream dispensers. There are case reports of myeloneuropathy involving the posterior and lateral columns (aka: subacute combined degeneration of the spinal cord) [50, 51] and a fatality [52] from inhalation of nitrous oxide from whip-cream dispensers.

Unfortunately, with the use of nitrous oxide in the emergency department, comes the potential for abuse. In 1995, there was a published case report of a hospital worker who had access to the hospital nitrous oxide supply and died from asphyxia due to unauthorized N_2_O inhalation [53]. Such cases highlight the importance of robust security procedures and equipment when storing and utilizing nitrous oxide.

## Conclusions

Although limited as solely an anesthetic agent, nitrous oxide remains a versatile and useful tool for treatment of pain and anxiety from the operating room to the dental office to the emergency department. Its efficacy and safety is well established, particularly in the pediatric emergency department, where its use alone or in combination with other agents can help facilitate performing painful and/or anxiety-provoking procedures such as the reduction of fractures and repair of lacerations.

Its use is limited by the ability to provide adequate ventilation/scavenging, as well as the ability to appropriately secure the agent and its delivery equipment to prevent abuse. As with any agent, there exists the potential for harm with its use. Notably, when used for PSA in the emergency department, these risks are low, but increase with increased duration of use, increased dose, concurrent use of other medications, and with complicating host factors.

Potential future applications of N_2_O, following further study, may include treatment of pain in EMS patients while still in the pre-hospital setting.
